# Angiopoietin‐like 4 production upon treatment with hypoxia and L‐mimosine in periodontal fibroblasts

**DOI:** 10.1111/jre.12649

**Published:** 2019-03-20

**Authors:** Klara Janjić, Alwina Schellner, Alexander Engenhart, Kurt Kernstock, Barbara Schädl, Andreas Moritz, Hermann Agis

**Affiliations:** ^1^ Department of Conservative Dentistry and Periodontology University Clinic of Dentistry Medical University of Vienna Vienna Austria; ^2^ Austrian Cluster for Tissue Regeneration Vienna Austria; ^3^ Ludwig Boltzmann Institute for Experimental and Clinical Traumatology Vienna Austria; ^4^ University Clinic of Dentistry Medical University of Vienna Vienna Austria

**Keywords:** ANGPTL4, HIF‐1alpha, hypoxia, spheroids

## Abstract

**Background and objective:**

A key factor in the modulation of angiogenesis as well as in bone resorption is angiopoietin‐like 4. However, the role of angiopoietin‐like 4 in periodontal tissue is unknown. Here, we hypothesized that hypoxia and the hypoxia mimetic agent L‐mimosine can induce the production of angiopoietin‐like 4 in periodontal fibroblasts.

**Methods:**

Human periodontal ligament fibroblasts (PDLF) were cultured in monolayer and spheroid cultures. The cultures were incubated in the presence of hypoxia or L‐mimosine. Angiopoietin‐like 4 mRNA and protein levels were measured by qPCR and ELISA, respectively. Also, the impact of Lipopolysaccharides of *E. coli* and *P. gingivalis*, interleukin (IL)‐1β and tumor necrosis factor (TNF)α was evaluated. Furthermore, we tested dependency on hypoxia‐inducible factor (HIF)‐1 activity by Western blotting for HIF‐1 and inhibitor studies with echinomycin. Potential autocrine effects were assessed by exposure of PDLF to recombinant angiopoietin‐like 4 in full length, C‐terminal and N‐terminal fragments. The impact on viability, DNA synthesis, alkaline phosphatase, and matrix mineralization was evaluated.

**Results:**

Both hypoxia and L‐mimosine elevated angiopoietin‐like 4 mRNA and protein levels in monolayer cultures of PDLF. HIF‐1 was elevated after both hypoxia and L‐mimosine treatment. LPS, IL‐1β, and TNFα did not modulate angiopoietin‐like 4 levels significantly. Addition of echinomycin in the cultures inhibited the production of angiopoietin‐like 4. In spheroid cultures of PDLF, the increase did not reach the level of significance at mRNA and protein levels. Angiopoietin‐like 4 in full length, C‐terminal, and N‐terminal fragments did not modulate viability, DNA synthesis, alkaline phosphatase, and matrix mineralization.

**Conclusion:**

Overall, we found that hypoxia and the hypoxia mimetic agent L‐mimosine can stimulate angiopoietin‐like 4 production in monolayer cultures of PDLF. This increase depends on HIF‐1 activity. Future studies will reveal how the modulation of angiopoietin‐like 4 in the periodontium contributes to periodontal disease and regeneration.

## 1 INTRODUCTION

The rising evidence on the capacity of hypoxia‐based regenerative strategies has intensified the research on the effect of hypoxia in periodontal tissues.^1^, ^2^ The current literature highlights that cells of oral tissues are potential targets for these strategies, including hypoxia pre‐conditioning and the application of hypoxia mimetic agents.^2^ However, the impact of hypoxia on periodontal cells is not yet fully understood. A key player in the response to hypoxia is angiopoietin‐like 4.^3^, ^4^, ^5^, ^6^ Angiopoietin‐like 4 belongs to the angiopoietin‐like proteins, which share similarities with the angiopoietin family.^3^ Angiopoietin‐like 4 is involved in the regulation of the processes underlying lipid and glucose metabolism and regeneration.^6^ It is expressed in a variety of tissues including adipose tissue, liver, pancreas, kidney, intestine, brain, placenta, skin, and blood.^7^ Expression was also confirmed in oral tissues, for example, the mandible in the early phase of regeneration.^8^ In vitro studies showed an increase of angiopoietin‐like 4 in mineralizing periodontal cells at day 14 of culture.^9^ Angiopoietin‐like 4 is increased under hypoxic conditions in non‐oral tissues.^10^ Also, hypoxia mimetic agents which stabilize hypoxia‐inducible factor (HIF)‐1 can upregulate angiopoietin‐like 4 in the non‐oral cell line HMEC‐1.^10^ It is possible that a similar mechanism is involved in periodontal tissue during healing processes.

Is angiopoietin‐like 4 an anabolic or catabolic factor? A broad spectrum of evidence has supported the notion that angiopoietin‐like 4 is involved in catabolic processes such as bone resorption.^11^, ^12^ It mediates the enhanced resorption activity of osteoclasts under hypoxic conditions.^12^ Interestingly, it is also elevated in degenerative diseases such as rheumatoid arthritis and osteonecrosis of the femoral head as well as in osteolytic tumors.^11^, ^13^, ^14^, ^15^ The fact that pro‐inflammatory factors can induce the expression of angiopoietin‐like 4 and that angiopoietin‐like 4 itself can mediate inflammatory disease suggests that it might also play a role in periodontal disease.^16^, ^17^, ^18^ The fact that HIF‐1 is found in gingivitis and periodontitis 19, 20 and that hypoxia and LPS can induce HIF‐1 synergistically suggest an involvement of angiopoietin‐like 4 in periodontal disease. However, a link between hypoxia, HIF‐1, and angiopoietin‐like 4 has not been established yet in periodontal cells.

We hypothesized that hypoxia and the hypoxia mimetic agent L‐mimosine can induce the production of angiopoietin‐like 4 in fibroblasts of the periodontal ligament (PDLF) involving HIF‐1 signaling. To test this hypothesis, we used monolayer PDLF and measured angiopoietin‐like 4 mRNA and protein levels in response to hypoxia and the hypoxia mimetic agent L‐mimosine. In addition, spheroid cultures of PDLF were applied to mimic the 3D environment as suggested for other tissues.^21^, ^22^ Tissue engineering methods using hypoxia‐treated cell‐derived spheroids provide a promising approach for oral tissue regeneration.^23^, ^24^, ^25^ Understanding the response of spheroids to hypoxia is thus of high relevance. To clarify the impact of pro‐inflammatory factors on angiopoietin‐like 4 production, we exposed PDLF to lipopolysaccharide (LPS) of *Porphyromonas gingivalis* and LPS of *Escherichia coli*, interleukin (IL)‐1β, and tumor necrosis factor (TNF)α. To reveal the involvement of HIF‐1, we performed Western blots for HIF‐1α and experiments in the presence of the inhibitor echinomycin. Furthermore, potential autocrine effects were tested by exposure of PDLF to recombinant angiopoietin‐like 4 in full length, C‐terminal, and N‐terminal fragments.

## 2 MATERIAL AND METHODS

### 2.1 Generation and expansion of fibroblasts of the periodontal ligament

Primary human PDLF were isolated from extracted third molars without any signs of inflammation if informed consent was given by the donors (1065/2013, Ethics Committee of the Medical University of Vienna, Vienna, Austria), following an established protocol.^26^ We included patients above 18 years, male and female. No quadrant was excluded. The periodontal ligament was scratched from the root surface and transferred into cell culture plates. Cell outgrowth and expansion were conducted in alpha‐minimal essential medium (alpha MEM, Sigma‐Aldrich, St. Louis, MO, USA) supplemented with 10% fetal bovine serum (FBS; Gibco, Thermo Fischer Scientific, MA, USA) and antibiotics (Thermo Fischer Scientific) and incubated at 37°C, 5% CO_2_, and 95% atmospheric moisture.

### 2.2 Monolayer culture

PDLF at 50 000 cells/cm^2^ were cultured in the presence of hypoxia or L‐mimosine at 1 mmol/L in alpha‐minimal essential medium supplemented with 10% FBS and antibiotics for 24 hours. The conditions were based on preliminary unpublished experiments and previous in vitro studies.^27^, ^28^ To establish hypoxic conditions we used an established protocol with minor modifications.^29^, ^30^, ^31^ The culture plates were placed into BD GasPak EZ Pouches (Becton, Dickinson and Company, Franklin Lakes, NJ, USA). The manufacturer confirms that the oxygen concentration rapidly decreases to a level of <1%. To verify the low oxygen levels an indicator was used as suggested by the manufacturer. Untreated PDLF cultured at 37°C and 5% CO_2_ under normoxic conditions (ambient O_2_ levels of 21%) served as normoxic control group. To assess the involvement of HIF‐1 signaling, experiments were performed in the presence of echinomycin at 1 μmol/L.

In indicated experiments, PDLF were treated with *E. coli* LPS at 0.1 μg/mL (InvivoGen, San Diego, CA, USA), *P. gingivalis* LPS at 1 μg/mL (InvivoGen), IL‐1β at 10 ng/mL (PeproTech Austria, Vienna, Vienna, Austria), or TNFα at 10 ng/mL (PeproTech Austria), with and without the presence of hypoxia or L‐mimosine in serum‐free medium. The concentrations of the factors were based on previous work.

Cells were subjected to RNA isolation, reverse transcription, qPCR analysis, and Western blotting. Culture supernatants were subjected to enzyme‐linked immunosorbent assay (ELISA). In the indicated experiments, cells were exposed to recombinant angiopoietin‐like 4 in full length (4487‐AN‐050), C‐terminal (3485‐AN‐050), and N‐terminal (8249‐AN‐050) fragments (all R&D Systems, MN, USA) at 100, 30, 10, and 3 ng/mL. The cells were then subjected to MTT assays, BrdU incorporation assays, alkaline phosphatase staining, and alizarin staining. Experiments for the MTT assay, alkaline phosphatase staining, and alizarin staining were performed in medium with 10% FBS. Experiments for proliferation were performed in serum‐free medium.

### 2.3 Spheroid culture

PDLF spheroids were generated using 3D Petri Dishes^®^ (Microtissues Inc., Providence, RI, USA). The 3D Petri Dishes^®^ were filled with agarose to produce molds. The agarose molds were then cooled and immersed in medium (Gibco, PAA). The molds were put into separate wells in the culture plates. As proposed by the manufacturer, the 75 μL cell suspension of 7 300 000 cells/mL was added. After PDLF had settled, the wells were filled with alpha‐minimal essential medium supplemented with 10% FBS and antibiotics. Once spheroid formation was completed after 24 hours, the cells were cultured in the presence of hypoxia or L‐mimosine at 1 mmol/L in alpha‐minimal essential medium supplemented with 10% FBS and antibiotics. Cell spheroids were subjected to RNA isolation, reverse transcription, and qPCR analysis. Culture supernatants were subjected to ELISA.

### 2.4 RNA isolation, reverse transcription, and quantitative polymerase chain reaction

Total RNA was isolated from PDLF with the RNeasy Plus Mini Kit (Qiagen, Hilden, NW, Germany) following the instructions of the manufacturer. Reverse transcription was performed with the High Capacity cDNA Reverse Transcription Kit (Applied Biosystems, Carlsbad, CA). Quantitative polymerase chain reaction was performed with the TaqMan^®^ Real‐Time PCR Master Mix (Applied Biosystems) and TaqMan^®^ assays (Applied Biosystems) for angiopoietin‐like 4 (Hs01101127_m1) and *Gapdh* (Hs02758991_g1). *Gapdh* was used as a reference gene. The relative mRNA levels were calculated by the ΔΔCt method.

### 2.5 ELISA

Culture supernatants of PDLF were subjected to ELISA for human angiopoietin‐like 4 by applying the human Angiopoietin‐like 4 DuoSet^®^ Elisa kit (R&D Systems Europe, Ltd. Abingdon, UK). Absorption measurements were performed as described by the manufacturer with the Synergy HTX multiplate reader (BiotTek, Winooski, Vermont, USA). Protein concentration of angiopoietin‐like 4 in the culture supernatant was calculated by the standard curve method.

### 2.6 Western blotting

Total cellular protein was isolated from PDLF cultured in monolayer and spheroid cultures as described above. Laemmli Sample Buffer (Bio‐Rad Laboratories GmbH, Vienna, Austria) was applied according to the manufacturer's protocol. Protein was then separated on SDS page. After transferring onto nitrocellulose membranes, detection was performed using the following primary antibodies (Thermo Fisher Scientific) to detect the target protein anti‐HIF‐1 α antibody (H‐206, Santa Cruz Biotechnology, Santa Cruz, CA, USA). Anti‐GAPDH (MA5‐15738, Thermo Fischer Scientific) was used to detect the reference protein GAPDH. The primary antibodies were then detected using the appropriate secondary antibody. Subsequently, chemiluminescence detection was performed with a ChemiDoc MP System (Bio‐Rad Laboratories, Inc. CA, USA).

### 2.7 Histology

Four percent of acid‐free neutral buffered formalin was used to fixate the spheroid cultures. The spheroids were then washed in water and dehydrated in a series of alcohol solutions. Consequently, the spheroids were embedded in paraffin. Sections of the samples were done using a rotary microtome. These sections were dried at 37°C. This process was followed by deparaffinization and rehydration steps. Then the sections were stained with haematoxylin and eosin as reported previously.^32^

### 2.8 MTT assay

Periodontal ligament fibroblasts in monolayer cultures in culture medium were exposed to recombinant angiopoietin‐like 4 in full length, C‐terminal, and N‐terminal fragments at 100, 30, 10, and 3 ng/mL for 24 hours. For the last 2 hours, the cells were incubated with 1 mg/mL MTT (3‐[4,5‐dimethythiazol‐2‐yl]‐2,5‐diphenyltetrazolium bromide) at 37°C for the last two hours of culture. The MTT solution was then removed and formazan crystals were solubilized using dimethyl sulfoxide. Optical density was measured with a photometer at 550 nm wavelength. Data were normalized to untreated PDLF (controls).

### 2.9 BrdU incorporation assay

To measure cell DNA synthesis as a marker for proliferation, PDLF in monolayers were cultured as described previously and labeled with bromodeoxyuridine (5‐Bromo‐2‐deoxyUridine (BrdU) Cell Proliferation ELISA, BrdU (colorimetric) Roche Diagnostics GmbH, Vienna, Austria) for the last two hours of exposure to angiopoietin‐like 4 in full length, C‐terminal, and N‐terminal fragments. The BrdU assay was performed as described by the manufacturer. Optical absorbance was measured at 450 nm wavelength. Data were normalized to untreated PDLF (controls).

### 2.10 Histochemical staining for alkaline phosphatase

Periodontal ligament fibroblasts in monolayer cultures were cultured as described previously in culture medium supplemented with 50 mmol/L L‐ascorbic acid and 10 mmol/L b‐glycerophosphate (Sigma‐Aldrich) for 7 days in the presence of recombinant angiopoietin‐like 4 in full length, C‐terminal, and N‐terminal fragments at 100, 30, 10, and 3 ng/mL. PDLF were fixed with neutral buffered formalin and incubated with the substrate solution containing Naphthol AS‐TR phosphate disodium salt and Fast Blue BB Salt (Sigma‐Aldrich). Staining intensity was quantified based on photometric assessment at 650 nm.

### 2.11 Alizarin red staining

Periodontal ligament fibroblasts were cultured in monolayers as described above and the medium was supplemented with 50 mmol/L L‐ascorbic acid and 10 mmol/L β‐glycerophosphate (Sigma‐Aldrich) for 14 days. PDLF were then fixed with 4% acid‐free neutral buffered formalin and stained with 0.5% alizarin red solution (Sigma‐Aldrich) at room temperature. Staining intensity was quantified based on photometric assessment at 450 nm.

### 2.12 Statistical analysis

IBM SPSS Statistics Version 23 (IBM Corporation, Armonk, NY, USA) was used to analyze the data. We performed Kruskal‐Wallis tests and Mann‐Whitney tests. The level of significance was set at *P *<* *0.05. Experiments were performed three times with two different donors, respectively. In experiments where cells were treated with angiopoietin‐like 4 in full length, C‐terminal fragments, N‐terminal fragments, *E. coli* LPS, *P. gingivalis* LPS, IL‐1β or TNFα, three different donors were used in two separately performed experiments. In all cases N = 6.

## 3 RESULTS

### 3.1 Hypoxia and L‐mimosine can increase angiopoietin‐like 4 in monolayer cultures of fibroblasts of the periodontal ligament

**Figure 1:**
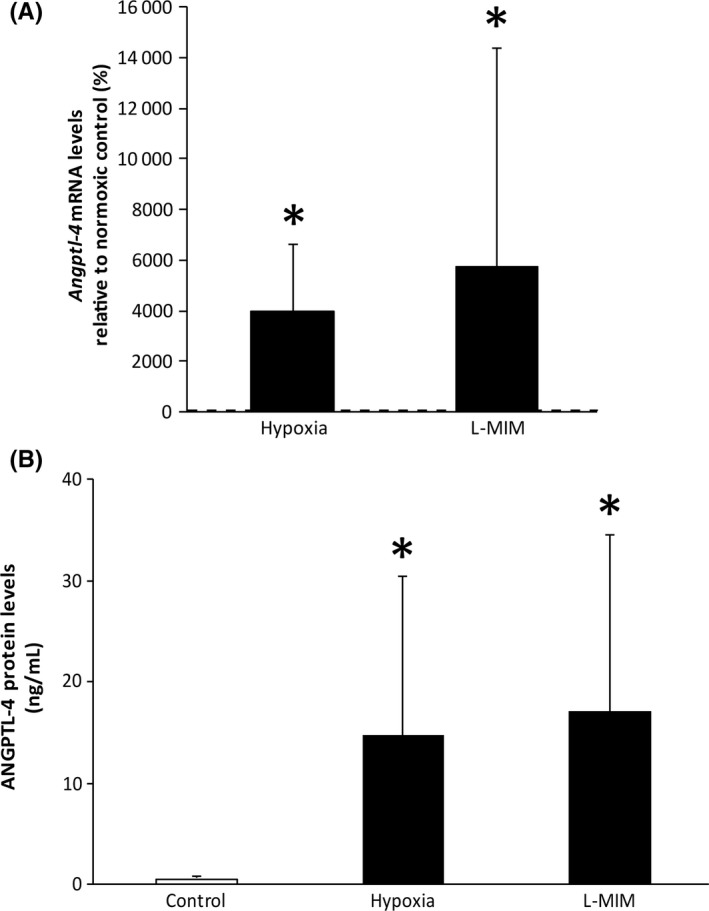
Hypoxia and L‐mimosine can increase angiopoietin‐like 4 in monolayer cultures of fibroblasts of the periodontal ligament. Human fibroblasts of the periodontal ligament (PDLF) in monolayer cultures were incubated in hypoxia or stimulated with L‐mimosine (L‐MIM) in medium with serum. Angiopoietin‐like 4 was measured at mRNA level (*Angptl4;* A) and protein level (ANGPTL4; B) using qPCR and ELISA, respectively. Bars represent mean + standard deviation, relative to the normoxic control. Experiments were performed three times with two different donors, respectively (N = 6). *P <* *0.05 vs control (dashed line; A, white bar; B)

We found that angiopoietin‐like 4 was increased at the mRNA level by hypoxia and the hypoxia mimetic agent L‐mimosine in PDLF (Figure 1). Hypoxia increased the mRNA levels 24‐fold in PDLF. L‐mimosine increased the mRNA levels 16‐fold. Also, at the protein level angiopoietin‐like 4 was stimulated by hypoxia and the hypoxia mimetic agent L‐mimosine. Hypoxia increased the protein levels 20‐fold in PDLF. L‐mimosine increased the protein levels 14‐fold. These data show that hypoxia and L‐mimosine increase angiopoietin‐like 4 in monolayer cultures of PDLF.

**Figure 2:**
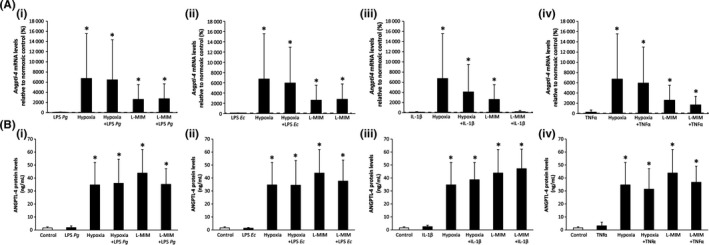
Angiopoietin‐like 4 in monolayer cultures of fibroblasts of the periodontal ligament stimulated with *P. gingivalis* lipopolysaccharide, *E. coli* lipopolysaccharide, *IL‐1*β*, and TNF*α in the presence of hypoxia or L‐mimosine. Human fibroblasts of the periodontal ligament (PDLF) in monolayer cultures were treated with *P. gingivalis* lipopolysaccharide (LPS Pg, A(i), B(i)), *E. coli* lipopolysaccharide (LPS Ec, A(ii), B(ii)), IL‐1β, A(iii), B(iii), TNFα, A(iv), B(iv), with and without the presence of hypoxia or L‐mimosine in medium without serum. Angiopoietin‐like 4 was measured at mRNA levels (*Angptl4; A*) and protein levels (ANGPTL4; B) using qPCR and ELISA, respectively. Bars represent mean + standard deviation, relative to the normoxic control. Experiments were performed twice with three different donors, respectively (N = 6). *P < 0.05 vs control (dashed line; A, white bar; B)

We further found that neither LPS from *P. gingivalis* nor *E. coli* modulated angiopoietin‐like 4 production at the mRNA or protein level with and without the presence of hypoxia or L‐mimosine. Also, IL‐1β and TNFα did not change the angiopoietin‐like 4 production both at mRNA and protein levels, although IL‐1β inhibited L‐mimosine‐induced mRNA production of angiopoietin‐like 4 (Figure 2). Overall, our data suggest that angiopoietin‐like 4 production in PDLF is regulated by hypoxia mediated signaling not involving pro‐inflammatory stimuli.

**Figure 3:**
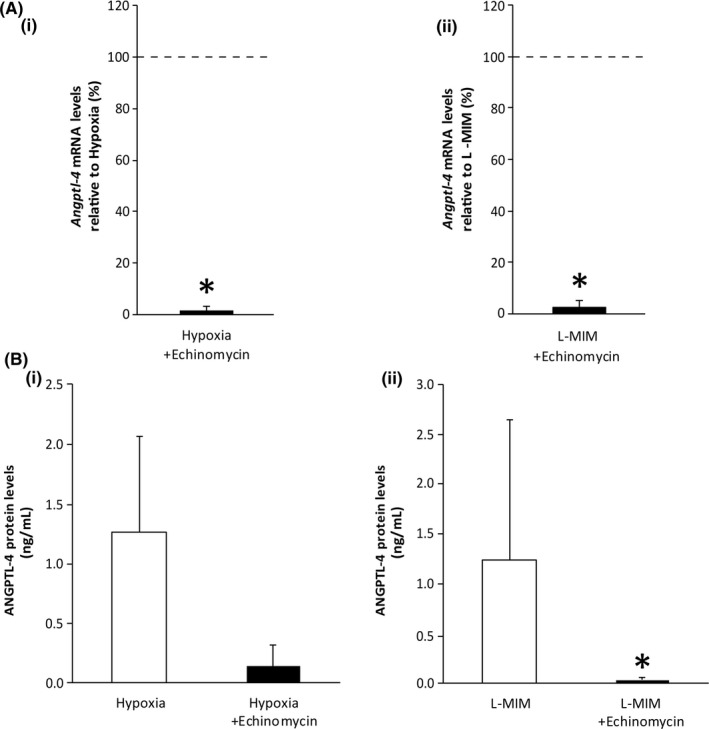
Hypoxia‐inducible factor‐1 is involved in the impact of hypoxia and L‐mimosine angiopoietin‐like 4 levels in fibroblasts of the periodontal ligament. Human fibroblasts of the periodontal ligament (PDLF) in monolayer cultures were incubated in hypoxia or stimulated with L‐mimosine (L‐MIM), with and without the presence of echinomycin in medium with serum. mRNA levels of angiopoietin‐like 4 (*Angptl4*; A (i‐ii)) and protein levels of angiopoietin‐like 4 (ANGPTL4; B(i‐ii)) were assessed by qPCR and ELISA, respectively. Bars represent mean + standard deviation, relative to the normoxic control. Experiments were performed three times with two different donors, respectively (N = 6). *P < 0.05 vs control (dashed line; A, white bar; B)

Echinomycin was added to the monolayer cultures of PDLF to reveal if HIF‐1 is involved in the effect of hypoxia and L‐mimosine on the production of angiopoietin‐like 4. Our data show that echinomycin decreases the mRNA levels of angiopoietin‐like 4 in the presence of hypoxia and L‐mimosine (Figure 3). This decrease was paralleled at protein level (Figure 3). The treatment with echinomycin reduced the protein levels in several samples below the detection limit. Overall, these data suggest that HIF‐1 is involved in the impact of hypoxia and L‐mimosine on angiopoietin‐like 4 production in PDLF.

### 3.2 Hypoxia‐inducible factor‐1 is involved in the impact of hypoxia and L‐mimosine on angiopoietin‐like 4 levels in fibroblasts of the periodontal ligament

**Figure 4:**
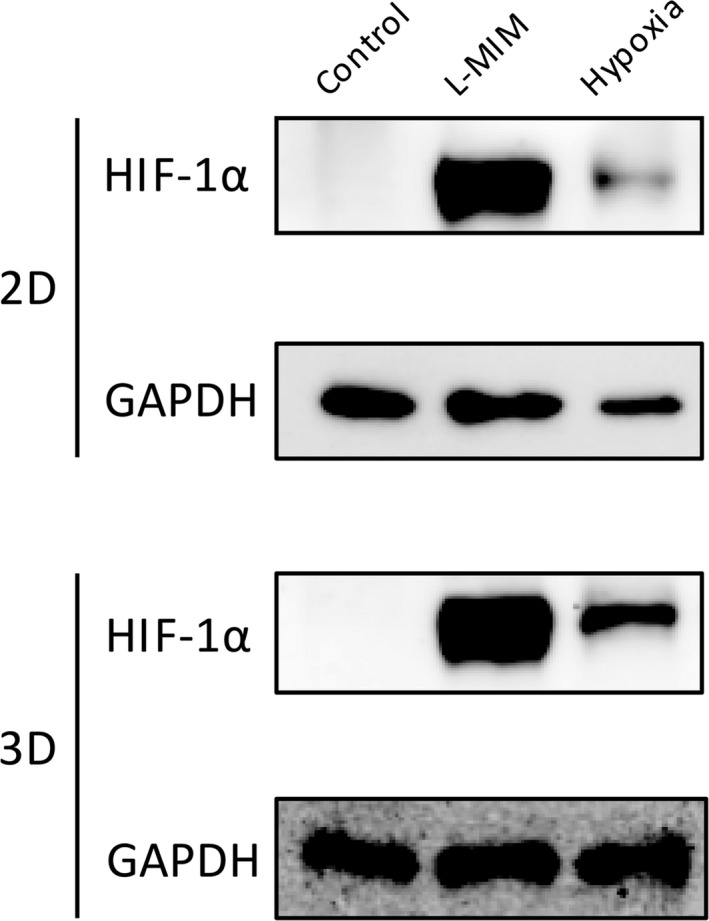
Hypoxia‐inducible factor‐1 is stabilized by hypoxia and L‐mimosine in fibroblasts of the periodontal ligament. Human fibroblasts of the periodontal ligament (PDLF) (2D) and spheroid cultures (3D) were incubated in hypoxia or stimulated with L‐mimosine (L‐MIM) in medium with serum. Western blotting for HIF‐1α was performed

Furthermore, we evaluated the impact of hypoxia and L‐mimosine on intracellular HIF‐1α levels by Western blotting (Figure 4). We found bands on the level of HIF‐1α upon exposure to L‐mimosine or hypoxia in both monolayer and spheroid cultures of PDLF.

### 3.3 Angiopoietin‐like 4 is expressed in spheroid cultures of fibroblasts of the periodontal ligament

**Figure 5:**
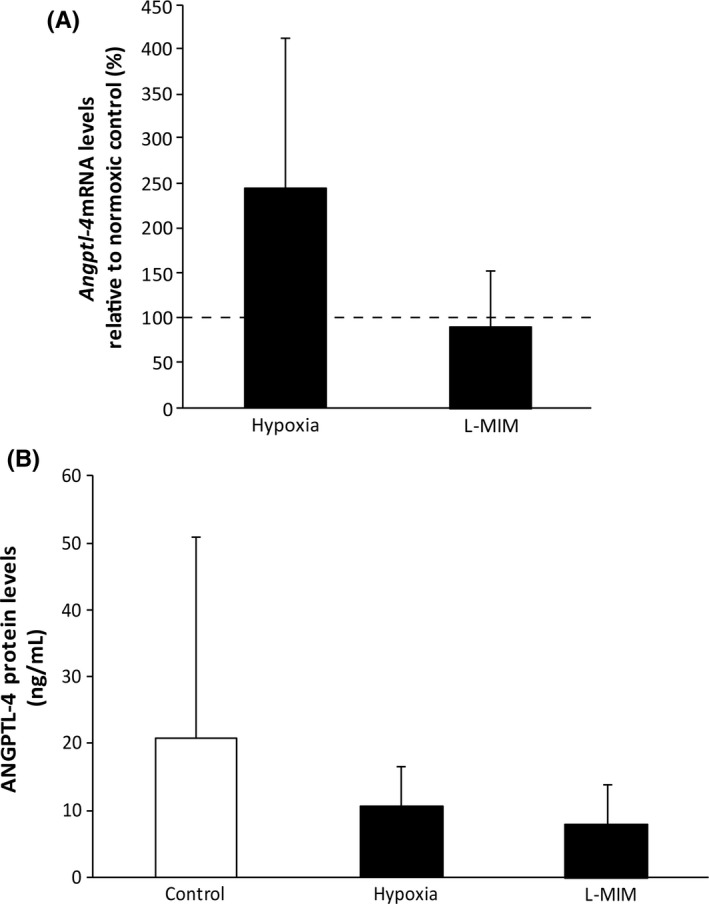
Angiopoietin‐like 4 is expressed in spheroid cultures of fibroblasts of the periodontal ligament. Human fibroblasts of the periodontal ligament (PDLF) in spheroid cultures were incubated in hypoxia or stimulated with L‐mimosine (L‐MIM) in medium with serum. Angiopoietin‐like 4 was measured at mRNA level (*Angptl4; A*) and protein level (ANGPTL4; B) using qPCR and ELISA, respectively. Bars represent mean + standard deviation, relative to the normoxic control. Experiments were performed three times with two different donors, respectively (N = 6). *P < 0.05 vs control (dashed line; A, white bar; B)

**Figure 6:**
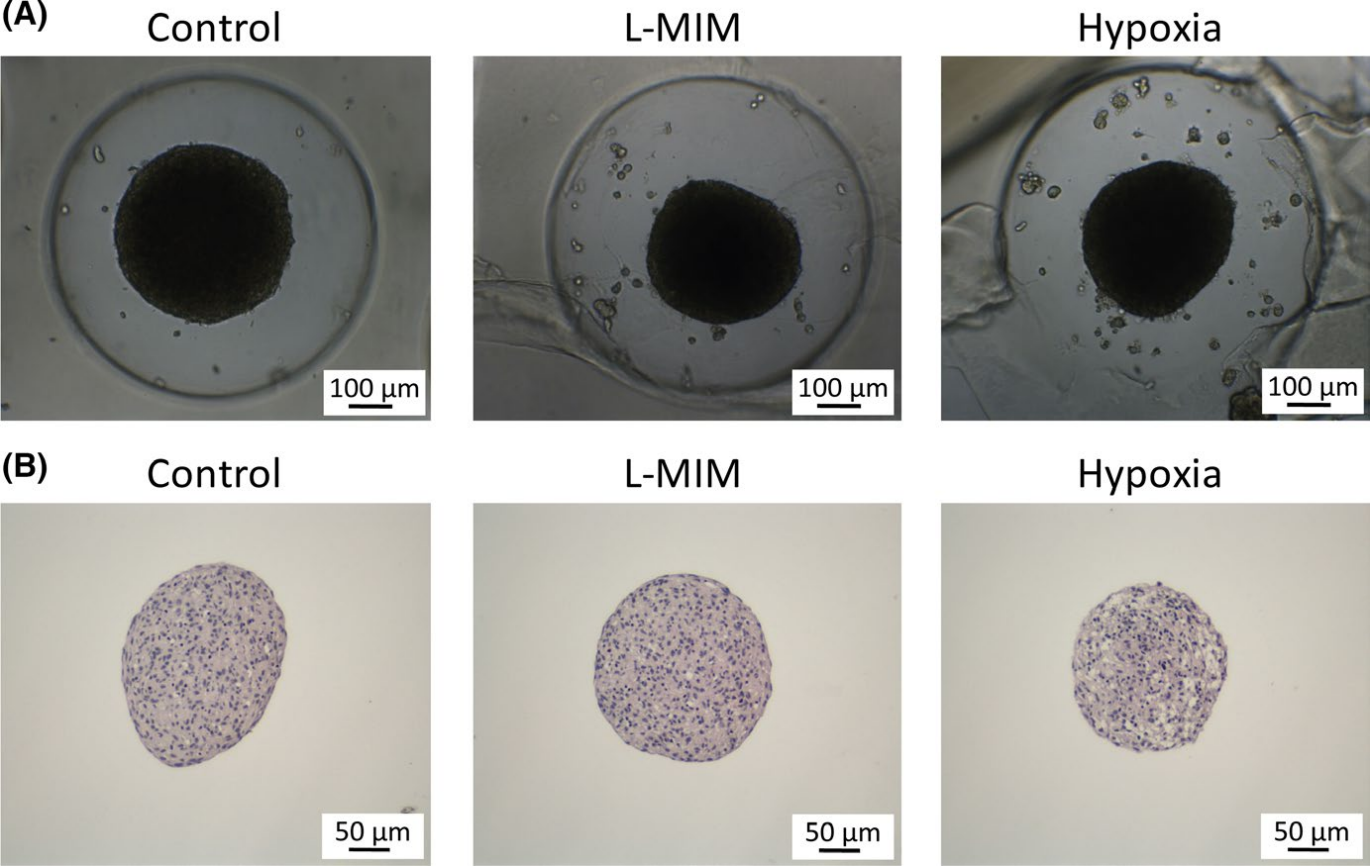
Images and histological sections of spheroid cultures of fibroblasts of the periodontal ligament in the presence of L‐mimosine and hypoxia. Spheroids of fibroblasts of the periodontal ligament were cultured in the presence of L‐mimosine (L‐MIM) at 1 mmol/L or hypoxia for 24 hours in medium with serum. Images of the cultures were taken using light microscopy (A), and histological sections were prepared. The images of the sections show hematoxylin and eosin staining of the spheroids (B)

Spheroid cultures of PDLF were used to mimic the periodontal tissue in vitro. Our results show that PDLF cultured in spheroids express angiopoietin‐like 4 at mRNA and protein levels (Figure 5). The levels in the untreated spheroids were higher than in the untreated monolayer cultures. However, the increase upon treatment with hypoxia or L‐mimosine did not reach the level of significance (Figure 5). No pronounced impact on spheroid morphology was observed when evaluating the spheroids after 24 hours of incubation and in the H&E staining (Figure 6). Overall, this suggests that the impact of hypoxia and L‐mimosine on angiopoietin‐like 4 production in PDLF spheroids was not as pronounced as in monolayer cultures.

### 3.4 Angiopoietin‐like 4 in full length, C‐terminal, and N‐terminal fragments does not dominantly modulate viability, DNA synthesis, alkaline phosphatase, and matrix mineralization

**Figure 7:**
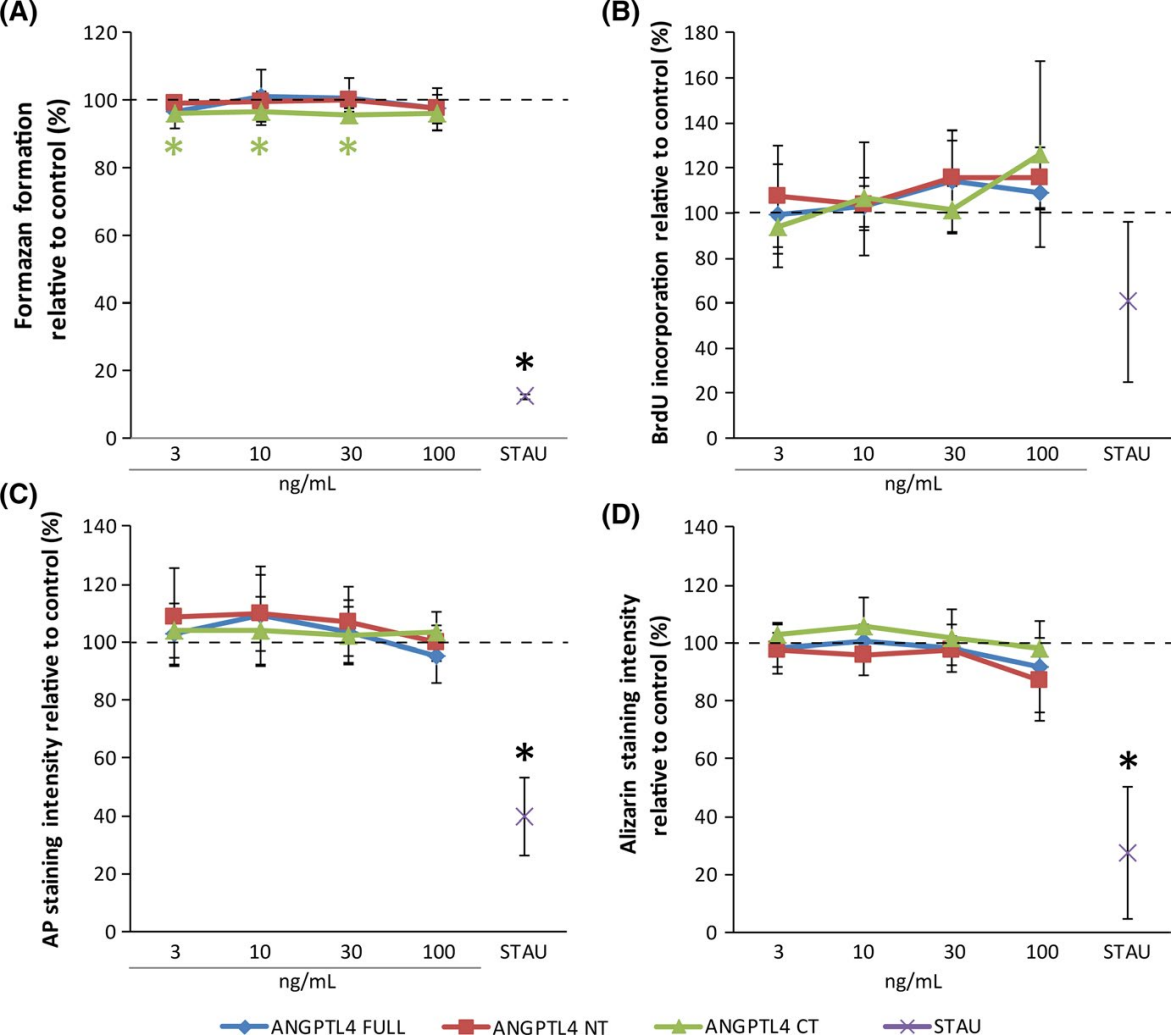
Angiopoietin‐like 4 full length, C‐terminal, and N‐terminal fragments do not dominantly modulate viability, proliferation, osteogenic differentiation, and matrix mineralization in monolayer cultures of fibroblasts of the periodontal ligament. Human fibroblasts of the periodontal ligament (PDLF) in monolayer cultures were incubated with recombinant angiopoietin‐like 4 full length, C‐terminal, and N‐terminal fragments at, 3, 10, 30, and 100 ng/mL in medium with serum. For the evaluation of alkaline phosphatase and matrix mineralization differentiation medium with serum was used. Viability (A) and DNA synthesis (B) was evaluated with MTT assay and BrdU incorporation assay, respectively. Viability experiments were performed in the presents of serum experiments on DNA synthesis were performed without the presence of serum. Alkaline phosphatase (C) and matrix mineralization (D) were evaluated based on histochemical staining (AP) and alizarin staining. Staurosporine (STAU) served as negative control. Experiments were performed twice with three different donors, respectively (N = 6) . *P < 0.05 vs control (dashed line)

To reveal the impact of angiopoietin‐like 4 in full length, C‐terminal, and N‐terminal fragments on viability, DNA synthesis, alkaline phosphatase, and matrix mineralization, we applied the MTT assay, BrdU incorporation assay, histochemical staining for AP, and alizarin staining (Figure 7). We found no distinct impact of all variants of angiopoietin‐like 4 in these assays. Only the C‐terminal fragment caused a slight decrease in formazan formation at 30‐3 ng/mL. Staurosporine showed a strong negative impact in all assays. Overall, our data suggest that no autologous impact on viability, DNA synthesis, alkaline phosphatase, and matrix mineralization differentiation can be expected.

## 4 DISCUSSION

A major signaling factor in the modulation of angiogenesis as well as in hypoxia‐induced bone resorption is angiopoietin‐like 4.^4^, ^6^, ^10^, ^11^, ^12^, ^33^ Furthermore, angiopoietin‐like 4 is involved in inflammation, lipid metabolism, and tissue regeneration.^3^, ^9^, ^13^, ^34^, ^35^ Together these roles make angiopoietin‐like 4 a potential target for therapeutic approaches in periodontology. However, the function of angiopoietin‐like 4 in periodontal tissue is still unclear. Here, we investigated the impact of hypoxia and the hypoxia mimetic agent L‐mimosine on angiopoietin‐like 4 expression in human primary PDLF. We found that PDLF can express angiopoietin‐like 4. Our results show that angiopoietin‐like 4 production is stimulated by hypoxia and the hypoxia mimetic agent L‐mimosine involving HIF‐1 signaling at mRNA and protein levels in monolayer cultures. These results are in line with our previous findings on the impact of hypoxia and L‐mimosine on angiopoietin‐like 4 in dental pulp‐derived cells.^36^ Similar findings have been reported for cell lines of non‐oral origin including tumor cells, adipocytes, and renal cells.^10^, ^37^ Yet, it seems that the response also depends on the differentiation status of the cells.^9^, ^38^ The fact that neither LPS nor IL‐1β, or TNFα can modulate angiopoietin‐like 4 suggests that angiopoietin‐like 4 production in PDLF is mainly regulated by hypoxia‐induced signaling. Only treatment with IL‐1β in combination with L‐mimosine led to an inhibition of angiopoietin‐like 4 mRNA production. The regulatory mechanism behind this effect is not clear yet. Future experiments have to evaluate whether intermingling pathways of immunomodulation and hypoxia are engaged. The concentrations of LPS, IL‐1β, and TNFα were chosen based on previous publications in oral fibroblasts. Although, we cannot exclude that lower or higher LPS, IL‐1β, and TNFα concentrations could modulate angiopoietin‐like 4 production. Therefore, dose dependence has to be analyzed in future experiments. Interestingly, the murine osteoblast cell line MC3T3 shows increased levels of angiopoietin‐like 4 upon IL‐1β stimulation. Therefore, it is possible that there are species‐ or tissue‐specific differences in the response.^17^ HIF‐1 was stabilized upon treatment with hypoxia or L‐mimosine. Echinomycin, a HIF‐1 signaling inhibitor, can reduce the expression levels of angiopoietin‐like 4, suggesting that HIF‐1 signaling is required for the increase of angiopoietin‐like 4. This finding is in line with our results on angiopoietin‐like 4 from dental pulp‐derived cells 36 and the impact of echinomycin treatment on vascular endothelial growth factor levels in dental pulp tissue.^32^ As echinomycin does not change the levels of HIF‐1α but inhibits the binding of the transcription factor to the DNA, we did not evaluate the protein levels of HIF‐1α in the presence of echinomycin.^39^ Interestingly, it seems that untreated PDLF cultured in 3D spheroid cultures show higher levels of angiopoietin‐like 4 than PDLF in untreated monolayer cultures. One can speculate that the increase in the protein levels might be explained by the higher numbers of cell. Since the number of cells per volume is 2‐fold higher in the spheroid cultures compared to the monolayer cultures, the 8‐fold higher angiopoietin‐like 4 levels in the spheroid group might not only be based on the higher cell count but could also be caused by the difference in culture conditions. In 3D spheroid cultures, we did not find an increase in angiopoietin‐like 4 upon treatment with hypoxia or L‐mimosine. These results are not in line with our results from the dental pulp.^36^ These results suggest that spheroid cultures of PDLF are not as sensitive to hypoxia and L‐mimosine treatment as monolayer cultures in regard to angiopoietin‐like 4 production. It is possible that cells in the core of the spheroid already reached the low cut‐off oxygen levels triggering angiopoietin‐like 4 expression. To assess this possibility, we also evaluated the intracellular HIF‐1α levels in spheroid cultures of PDLF. Interestingly, we found elevated HIF‐1α levels in spheroid cultures under hypoxic conditions and L‐mimosine treatment but not in the control group. Therefore, oxygen levels in the control group did not reach the level required for substantial HIF‐1α stabilization. It is thus possible that there are differences in the response of dental pulp‐derived cells and PDLF to the conditions in spheroid cultures with regard to ANGPTL4. The mechanism however remains to be determined in future studies.

Angiopoietin‐like 4 has a key role in a broad spectrum of osteolytic diseases including rheumatoid arthritis, osteoarthritis, bone cancer, and osteoporosis.^11^ Angiopoietin‐like 4 was also expressed in experimental periodontitis.^11^ Therefore, there is an interest in the development of therapeutic approaches that target angiopoietin‐like 4 to prevent bone resorption and improve regeneration. Currently however, the knowledge on the role of angiopoietin‐like 4 in periodontal disease is very limited. Thus, future research is required to reveal the role of angiopoietin‐like 4 in periodontal health and disease. Due to the complex mechanisms involved in angiopoietin‐like 4 production and function, the past decade of research on angiopoietin‐like 4 has left many unanswered questions. Therefore, requiring much effort to unravel this mystery in periodontal tissues.^6^ However, we can learn from other fields of research, which have revealed several of the involved mechanisms. For instance, angiopoietin‐like 4 is proteolytically processed and releases the C‐terminal fibrinogen‐like domain after proteolytic cleavage.^6^, ^40^, ^41^ Although the function of the angiopoietin‐like 4 C‐terminal fragment has not yet been clarified, previous studies have shown that hypoxia and L‐mimosine can modulate the proteolytic activity.^26^, ^42^, ^43^ In particular, the reduction of the plasminogen activation capacity was reported in fibroblasts of the gingiva and the periodontal ligament.^26^ Hypoxia and hypoxia mimetic agents such as L‐mimosine can therefore not only increase angiopoietin‐like 4 expression but may also induce proteolytic processing in the periodontium.

To reveal potential autologous effects of angiopoietin‐like 4, we treated periodontal cells with angiopoietin‐like 4 full length, C‐terminal, and N‐terminal fragments. We found that all three variants of angiopoietin‐like 4 did not modulate DNA synthesis, an indicator of cell proliferation. Nor did the three variants of angiopoietin‐like 4 modulate alkaline phosphatase and matrix mineralization activity, both indicators of osteoblastic differentiation. Interestingly, angiopoietin‐like 4 did increase proliferation and osteogenic differentiation in Saos2 cells.^12^ One can argue that it is unclear if PDLF express the angiopoietin‐like 4 receptor. However, angiopoietin‐like 4 is currently considered an orphan ligand as no specific receptor has been found so far.^11^ Therefore, future research needs to clarify the exact mechanism by which cells respond to the presence of angiopoietin‐like 4. Our data suggest that the produced angiopoietin‐like 4 can increase the anabolic activity of osteoblasts whilst not showing autologous effects. However, angiopoietin‐like 4 is also a driving force with multiple roles in the catabolic activities in osteolytic diseases.^11^

As angiopoietin‐like 4 mediates the enhanced bone resorption by osteoclasts under hypoxic conditions and periodontal disease provides a hypoxic micro‐environment it might be possible that angiopoietin‐like 4 can contribute to the catabolic activity in periodontal tissue.^12^ It was reported that hypoxia leads to an increase of angiopoietin‐like 4 in osteoclasts, thus stimulating bone resorption activity.^12^ Similar mechanisms might be involved in root resorption and tooth trauma. Inhibition of angiopoietin‐like 4 by specific antibodies might help to hamper this catabolic feedback mechanism. However, also potential positive involvement of angiopoietin‐like 4 during tooth development and tooth movement in orthodontic therapy needs to be considered. Therefore, a broad spectrum of fields in dentistry might profit from further research on the role of angiopoietin‐like 4 in periodontal tissue.

Overall, we found that hypoxia and L‐mimosine can increase angiopoietin‐like 4 expression in monolayer cultures of PDLF. Our inhibitor studies suggest that the underlying mechanism involves HIF‐1 activity. Future in vivo studies need to reveal the role of angiopoietin‐like 4 in periodontal health and disease.
